# Bilateral subdural hematomas and retinal hemorrhages mimicking nonaccidental trauma in a patient with D‐2‐hydroxyglutaric aciduria

**DOI:** 10.1002/jmd2.12188

**Published:** 2020-11-20

**Authors:** Ester Perales‐Clemente, Angela L. Hewitt, April L. Studinski, Jan‐Mendelt Tillema, William J. Laxen, Devin Oglesbee, Arne H. Graff, Piero Rinaldo, Brendan C. Lanpher

**Affiliations:** ^1^ Biochemical Genetics Laboratory, Department of Laboratory Medicine and Pathology Mayo Clinic Rochester Minnesota USA; ^2^ Department of Child Neurology Mayo Clinic Rochester Minnesota USA; ^3^ Departments of Pediatric and Adolescent Medicine and Family Medicine Mayo Clinic Rochester Minnesota USA; ^4^ Department of Clinical Genomics Mayo Clinic Rochester Minnesota USA

**Keywords:** D‐2‐hydroxyglutaric aciduria, nonaccidental trauma, retinal hemorrhages, shaken baby syndrome, subdural hematomas, urine organic acids

## Abstract

**Introduction:**

Nonaccidental trauma (NAT) is considered when pediatric patients present with intracranial injuries and a negative history of an accidental injury or concomitant medical diagnosis. The evaluation of NAT should include the consideration of possible medical causes including coagulation, hematologic, metabolic and other genetic disorders, as well as witnessed and unwitnessed accidental injuries.

**Case Presentation:**

We present a 7‐month‐old male with spells and incidental findings of bilateral subdural hematomas, retinal hemorrhages, and secondary macrocephaly, leading to investigation for NAT. Biochemical analysis showed excretion of a large amount of D‐2‐hydroxyglutaric in urine consistent with a biochemical diagnosis of D‐2‐hydroxyglutaric aciduria, a rare neurometabolic disorder characterized by developmental delay, epilepsy, hypotonia, and psychomotor retardation. None of these symptoms were present in our patient at the time of diagnosis. Molecular genetic testing revealed a pathogenic splice site variant (c.685‐2A>G) and a variant of uncertain significance (c.1256G>T) with evidence of pathogenicity in the *D2HGDH* gene, consistent with a molecular diagnosis of D‐2‐hydroxyglutaric aciduria type I (OMIM #600721).

**Conclusion:**

Since several metabolic disorders, including D‐2‐hydroxyglutaric aciduria type I, can present solely with symptoms suggestive of NAT (subdural and retinal hemorrhages), an early metabolic evaluation by urine organic acid analysis should be included in clinical protocols evaluating NAT. A methodical and nonjudgmental approach coordinated between pediatricians and metabolic specialists is also necessary to ensure that rare genetic conditions are not overlooked to prevent devastating social, legal, and financial consequences of suspected child abuse.

**Synopsis**

D‐2‐hydroxyglutaric aciduria type I is a rare disorder which may present initially with only subdural hematomas and retinal hemorrhages mimicking nonaccidental trauma and no other symptoms of a degenerative neurodevelopmental disorder.

## 1 INTRODUCTION

It has been estimated that 674 000 children were maltreated in the USA during 2017, from which 1720 children died from abuse and neglect (January 28).^1^ Nonaccidental trauma (NAT) is defined by an injury to the skull or intracranial contents of an infant or child younger than 5 years old, usually as a result of intentional, abrupt impact, and/or violent shaking. It is estimated that NAT is a primary cause of death and disability in children affected by abuse.^2^ Physicians play a crucial role in the recognition and diagnosis of NAT, which remains a challenge in many practice settings due to provider bias, preconceptions, and failure to recognize an acute presentation as possible abuse. Differential diagnoses need to be considered in every abuse evaluation to avoid unintended consequences including: increased parental stress, compromised doctor‐patient relationship, extended hospitalization with corresponding increased healthcare costs, and social and legal consequences. Multiple genetic conditions like bile transport defects,^3^ osteogenesis imperfecta,^4^ Menkes syndrome,^5^ and glutaric aciduria type I (GA‐I)^6^ among others,^7^ can mimic child abuse and should be excluded via laboratory testing as appropriate in order to prevent potentially devastating consequences for both patients and their families, as well as informing treatment and recurrence risk implications for genetic diagnoses.

Cerebral organic acidurias are a group of organic acidurias characterized by progressive neurological symptoms of ataxia, epilepsy, myoclonus, extrapyramidal symptoms, metabolic stroke and macrocephaly, which usually have a late onset presentation.^8^ This group of disorders include among others: glutaric aciduria type I and 2‐hydroxyglutaric acidurias. These disorders accumulate odd‐chain dicarboxylic acids (D‐2‐hydroxyglutaric acid, L‐2‐hydroxyglutaric acid, 3‐hydroxyglutaric acid, and glutaric acid) as pathological compounds in the brain, causing toxic effects that lead to progressive neurodegeneration. The 2‐hydroxyglutaric acidurias are a heterogeneous group of cerebral organic acidurias classified as D‐2‐hydroxyglutaric aciduria, L‐2‐hydroxyglutaric aciduria, and combined D, L‐2‐hydroxyglutaric aciduria that lead to neurological impairment at a young age, although affected individuals may appear normal at birth. Accumulation of D‐2‐hydroxyglutarate (D‐2HGA) and/or L‐2‐hydroxyglutarate (L‐2HGA) in body fluids are the biochemical hallmarks of these disorders.^9^ D‐2‐hydroxyglutaric aciduria is further classified in two groups: type I and type II. D‐2‐hydroxyglutaric aciduria type I (OMIM #600721) is an autosomal recessive disorder caused by pathogenic variants in *D2HGDH* on chromosome 2q37.3, which encodes for D‐2‐hydroxyglutarate dehydrogenase (D‐2‐HGDH) (EC1.1.99). D‐2‐HGDH deficiency is clinically characterized by developmental delay, epilepsy, and hypotonia as primary features. D‐2‐hydroxyglutaric aciduria type II (OMIM #600721) is an autosomal dominant disorder caused by gain‐of‐function variants in *IDH2* on chromosome 15q26, with symptoms appearing in the first 2 years of life and characteristically more severe than in type I, including cardiomyopathy. Macrocephaly, visual problems and facial dysmorphism can be seen in both forms as the disease evolves. Both gene defects result in accumulation of D‐2HGA in urine, plasma and CSF,^10^ so the distinction between them can only be achieved through molecular analysis of the genes involved in these conditions.

As mentioned above, patients with glutaric aciduria type 1 may suffer acute subdural hemorrhages, including retinal hemorrhages after minor head trauma, simulating NAT. In this study, we report an infant with bilateral subdural hematomas and retinal hemorrhages in whom NAT was initially investigated. Surprisingly, subsequent biochemical and molecular genetic analyses revealed a diagnosis of D‐2‐hydroxyglutaric aciduria type I, another cerebral organic aciduria described by Chalmers et al. in 1980.^11^ This case highlights the importance of evaluating urine organic acids as a screening test in infants suspected of NAT.

## 2 CASE PRESENTATION

A 7‐month‐old previously healthy male (Minnesota newborn screening negative) presented in the emergency room with concerns for head injury and loss of consciousness, after falling backwards while sitting on a carpeted floor. As mom picked him up, he abruptly stopped crying, and his eyes turned upwards as if staring. He had bilateral lower extremity tremulousness lasting about 5 seconds, and then “went limp.” Emergency medical services were notified. He “snapped out of it” when transported to a cold hallway 1 or 2 minutes later, but was sleepy.

**FIGURE 1:**
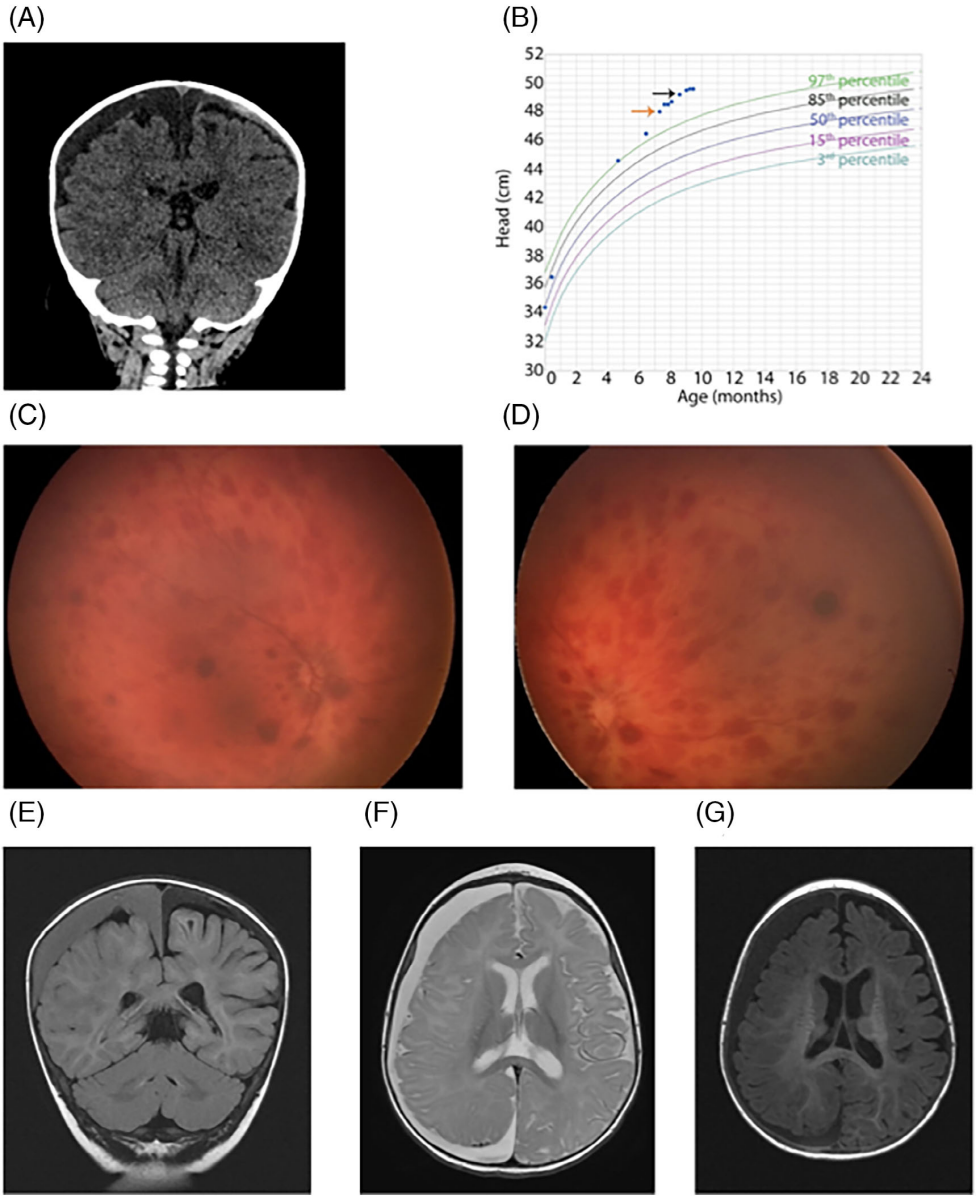
Clinical presentation. A, Computed tomography (CT) head: bilateral subdural hemorrhages of varying hypodensities overly both hemispheres; right brain hypodensity is dark grey, suggesting partial breakdown of chronic blood products; left brain hypodensity has light grey to white overlying dark grey, suggesting an acute on chronic subdural bleed. B, Head circumference measurements during time reported: admission (age 7 months, orange arrow) and second episode (age 9 months, black arrow). C and D, Dilated ophthalmology examination: multilayered intraretinal hemorrhages were observed in the posterior poles of both the right (C) and left (D) eyes. E‐G, MRI brain confirmed bilateral subdural hematomas of different ages across imaging sequences including T2 Flair coronal (E), T2 axial (F), and T1 axial (G). The right convexity subdural hematoma measured 14 mm maximum radial diameter (previously 11 mm on head CT); left subdural hematomas had distinctly different signal characteristics from each other, confirming differing ages. No other brain abnormalities were identified

In the emergency department, he had two episodes of nonbloody, nonbilious emesis. Initial screening labs, including complete blood count (CBC) and comprehensive metabolic panel (CMP: potassium, sodium, chloride, bicarbonate, anion gap, blood urea nitrogen, creatinine, calcium, protein, albumin, aspartate aminotransferase, alkaline phosphatase, alanine aminotransferase, and bilirubin) with glucose were unremarkable. Physical examination did not show any signs of trauma. Computed tomography (CT) head scan showed multiple bilateral subdural hemorrhages of different chronicities (Figure 1A). Pediatric neurosurgery reviewed his head CT and examined him in the ED. Given his normal exam, they referred to neurology and child protective services teams while continuing to follow his evaluations. The neurologic exam (including motor examination and reflexes) was normal, other than an increased orbitofrontal head circumference of 47.5 cm. There were no signs of abuse, other than a slightly erythematous area over the right occiput consistent with his reported fall. Routine EEG exam was negative for potentially epileptogenic activity. However, it did show decreased amplitude over the right frontotemporal head region during wakefulness, with sleep spindle activity also decreased over the right hemisphere, and age appropriate posterior dominant rhythm. No bone fractures or cervical spine ligamentous injury were found on a skeletal survey. Ophthalmology exam showed bilateral multilayered intraretinal hemorrhages in the posterior poles (Figure 1C,D).

The patient was the first child of nonconsanguineous parents, with a complicated social history including previously suggested risk factors for abuse.^12^ Father was in a correctional facility with a history of alcohol abuse. Mother had a history of attention‐deficit/hyperactivity disorder (ADHD), anxiety, post‐traumatic stress disorder (PTSD), and bipolar disorder. Per records, pregnancy was complicated by tobacco and tetrahydrocannabinol use, but a meconium drug screen at birth was negative. Due to the combination of subdural and retinal hemorrhages concerning for NAT, child protection protocols were initiated.

The differential diagnosis for bilateral subdural hematomas in a 7‐month‐old include: NAT, rebleed of a prior subdural collection (neomembrane), bleeding/clotting disorder, benign subdural hemorrhages of childbirth, and metabolic disorders such as glutaric aciduria type I or Menke's disease. Review of the growth chart showed rapid progression of macrocephaly, with head circumference growing from 46.5 cm (18.31 in., 99.06 percentile, Z = 2.35) to 48 cm (18.9 in., 99.91 percentile, Z = 3.12) in 3 weeks (Figure 1B, orange arrow). Hematological assays to evaluate for a bleeding disorder were essentially normal, with normal coagulation studies and slightly elevated fibrinogen = 0.68 mcg/mL (0‐0.5). D‐Dimer units = 340 ng/mL (0‐250) and soluble fibrin 25 mcg/mL (0‐7.9) were elevated as expected for a recovering brain bleed. Brain MRI, performed 2 days after admission, confirmed bilateral subdural hematomas of different ages with slight midline shift to the left (Figure 1E‐G).

### 2.1 Diagnosis

Urine organic acids were obtained to evaluate the possibility of glutaric aciduria type I given the findings of macrocephaly and NAT. Unexpectedly, analysis by gas chromatography/ mass spectrometry revealed markedly elevated excretions of 2‐hydroxyglutaric acid (2HGA, 1098 μg/mg creatinine; normal reference interval < 20) and 2‐hydroxy glutaric lactone (2HGL, 162 μg/mg creatinine; normal reference interval undetectable). 2HGA is an optically active acid, chromatographic separation of the enantiomers (D‐2‐HGA and L‐2‐HGA) confirmed the excretion of the D isomer in the patient's urine consistent with a biochemical diagnosis of D‐2‐hydroxyglutaric aciduria, which is a rare inherited neurometabolic disorder with a wide clinical spectrum. Some patients are asymptomatic, while others exhibit developmental delay, epilepsy, hypotonia, cardiomyopathy, and dysmorphic features. None of these clinical manifestations were present in our patient at biochemical diagnosis. Furthermore, plasma amino acids were essentially normal. Total and free serum carnitine levels were normal, and serum acylcarnitine profile was also normal. To rule out a possible low excretor phenotype of glutaric aciduria type I in our patient, urine acylcarnitines were analyzed. The urine excretion of glutarylcarnitine was within normal range (0.29 mmol/mol creatinine; reference interval <1.54 mmol/mol creatinine), ruling out possible comorbidity with glutaric aciduria type I.

In order to distinguish between D‐2‐hydroxyglutaric aciduria type I and type II, a hydroxyglutaric aciduria sequencing panel and copy number variant detection (prevention genetics) was performed analyzing: *D2HGDH*, *IDH2*, *L2HGDH*, and *SLC25A1*. The biochemical diagnosis of D‐2‐hydroxyglutaric aciduria was confirmed by molecular analysis. DNA sequencing revealed compound heterozygous variants in *D2HGDH*: a pathogenic splice site variant designated c.685‐2A>G, and a variant of uncertain significance c.1256G>T (p.Arg419Leu). These results were consistent with a molecular diagnosis of D‐2‐hydroxyglutaric aciduria type I.

### 2.2 Follow‐up

Two months after the first episode, our patient again presented to the emergency department with decreased level of consciousness or “spell” after hitting the left side of his head on a chair. Repeat head CT showed bilateral low density subdural fluid collections which had increased in size since the prior exams without findings of acute hemorrhage, compatible with chronic subdural hematomas or hygromas. The head circumference had further increased 1.5 cm since the last episode: 49.5 cm (19.49 in., 99.98 percentile, Z = 3.58) (Figure 1B, black arrow). Repeat skeletal survey revealed no new concerns. Repeat fundus examination showed improving bilateral intraretinal hemorrhages in the posterior poles, more in the left than the right eye. Repeat urine organic acids showed again that excretions of 2HGA (1178 mmol/mol creatinine; normal reference interval <20) and 2HGL (124 mmol/mol creatinine; normal reference interval undetectable) were markedly elevated, in the absence of other unusual organic acids, consistent with the current diagnosis. A routine EEG did not show any potential epileptic activity or focal slowing, and the posterior dominant rhythm was age appropriate at 6 Hz. An echocardiogram showed patent foramen ovale. He was again evaluated by our child protective services team, who agreed symptoms remained consistent with the diagnosis of D‐2‐hydroxyglutaric aciduria type I. It was recommended he start treatment with riboflavin supplementation (100 mg daily).

He was evaluated in neurometabolic and neurology clinics at age nine and a half months for outpatient follow‐up where he demonstrated mild gross motor delay. His mother reported increasingly frequent abrupt onset staring spells with unresponsiveness concerning for seizures. There was no associated eye deviation or abnormal movements. The spells occurred about once per week. Yet, he continues to gain skills and is a social, happy baby who smiles, babbles, and makes good eye contact. Muscle tone, strength, reflexes, and extraocular movements are also normal. His anterior fontanelle is largely patent at 2 cm × 1 cm, with an orbitofrontal head circumference of 50 cm. His last evaluation was at age 12 months. He continues to gain skills in all development domains. The frequency of spells decreased to about once per month, so EEG monitoring was not repeated. Repeat MRI showed resolving subdural hygromas. While this is positive, it is difficult to make any conclusions regarding riboflavin treatment in a single patient treated for only 2 1/2 months. The efficacy of riboflavin supplementation for D‐2‐hydroxyglutaric aciduria patients remains unknown.

## 3 DISCUSSION

The clinical presentation of D‐2‐hydroxyglutaric aciduria varies from neonatal onset of severe seizures, lack of psychomotor development and early death, to mild developmental delay or no symptoms.^13^ This clinical spectrum has been attributed to the existence of two types of D‐2‐hydroxyglutaric acidurias: type I caused by pathogenic variants in *D2HGDH* gene (OMIM# 600721) and type II caused by gain‐of‐function variants in *IDH2* gene (OMIM# 613657). Urine organic acids in our patient revealed marked elevation of 2‐hydroxyglutaric acid and 2‐hydroxyglutaric lactone. Supplementary studies of chromatographic separation of the enantiomers (D‐2‐HGA and L‐2‐HGA) specifically revealed the excretion of the D‐isomer consistent with a biochemical diagnosis of D‐2‐hydroxyglutaric aciduria. The specific subtype was identified as type I by molecular genetic testing showing two heterozygous variants in *D2HGDH*: a pathogenic splice site variant (c.685‐2A>G), and a variant of uncertain significance (c.1256G>T). Homozygosity for c.685‐2A>G, has been described previously in two clinically asymptomatic siblings with markedly elevated D‐2‐hydroxyglutaric aciduria. Fibroblast mRNA analysis indicated that the variant, c.685‐2A>G, altered normal mRNA splicing leading to nonsense‐mediated decay.^14^ The variant c.1256G>T is predicted to result in the amino acid substitution p.Arg419Leu. This variant has not been previously reported in the literature. However, a different homozygous substitution of the same amino acid (c.1256G>A, p.Arg419His) has been reported in a patient with D‐2‐hydroxyglutaric aciduria. The D2HGDH enzyme activity was reduced in the patient's fibroblasts compared with control cell lines (39 pmol/h mg; control cell lines: 247‐667 pmol/h mg) confirming the pathogenicity of the variant.^10^ Thus, following current ACMG standards and guidelines, the variant, c.1256G>T, falls into the category, PM5: a novel missense amino acid change occurring at the same position as another pathogenic missense change, which is considered as moderate evidence of pathogenicity. Currently, there is no effective treatment for D‐2‐hydroxyglutaric aciduria. Since the enzyme D2HGDH belongs to a family of enzymes that use flavin adenine dinucleotide (FAD) as a cofactor, riboflavin supplementation (vitamin B2, precursor of FAD) might be beneficial. Some studies suggest it is effective for L‐2‐hydroxyglutaric aciduria patients.^15^

While the biochemical and molecular diagnoses were in agreement, the clinical presentation of this patient differed from the majority of D‐2‐hydroxyglutaric aciduria type I patients previously described. Typical symptoms include developmental delay, epilepsy, hypotonia, and psychomotor retardation with a mean age of onset in the first year of life.^9^ None of these symptoms were present in our patient at the time of diagnosis. NAT was initially considered in our patient, who presented with macrocephaly, bilateral subdural hematomas, and retinal hemorrhages. Infants and toddlers with NAT injuries frequently do not exhibit macrocephaly at presentation, but rather present with the combination of subdural hematoma, retinal hemorrhage and encephalopathy. This is attributed to an injury mechanism in which bridging veins tear secondary to trauma. However, the differential diagnoses include coagulation, hematologic, and metabolic disorders.

The classic metabolic condition that mimics NAT is glutaric aciduria type I, a progressive neurodegenerative inborn error of metabolism that typically manifests acutely in infants with illnesses. Macrocephaly is usually present in the neonatal period or shortly after birth, before the first signs of neurological disease.^16^ Patients with GA‐I are predisposed to experience acute subdural hemorrhages after minor trauma,^17^ but some also show brain abnormalities such as fronto‐parietal brain atrophy or an open opercula on neuroimaging helping in the final diagnosis.^18^ Retinal hemorrhages are also described in GA‐I.^19^ Parents of children with GA‐I have been unjustly accused of child abuse following the identification of chronic or acute subdural hematoma in their children.^6^ The biochemical diagnosis is usually established by detection of high excretion of glutaric acid, 3‐hydroxyglutaric acid in urine organic acids, and glutarylcarnitine (C5DC) in plasma acylcarnitine analysis. Since C5DC can be detected by tandem mass spectrometry, and early treatment has been proved to be effective, GA‐I was included in national newborn screening (NBS) programs. In the largest cohort of early diagnosed GA‐I patients, 94 individuals had confirmed diagnosis of GA‐I, of whom 87 were identified through NBS. Four had false negative results.^20^ These four patients were later identified after onset of dystonia and encephalopathic crisis. All them had low excretor phenotype; patients who excrete low amounts of glutaric acid in urine and have low levels of plasma/DBS C5DC may be overlooked. Thus, the excretion of glutarylcarnitine in urine is the preferred biomarker to identify low excretors GA‐I patients when results are inconclusive for urine organic acid and plasma acylcarnitine profiles.^21^ Since our patient showed normal levels of glutaric and 3‐hydroxyglutaric acids in urine, and normal levels of C5DC in plasma acylcarnitines, urine acylcarnitines were performed to evaluate the excretion of glutarylcarnitine in urine, which was normal in our patient, excluding the possibility of a low excretor phenotype, and a second inborn error of metabolism in this patient.

Subdural hematomas are not specific for any metabolic condition. They can be caused by a severe head injury (acute and subacute) or by less severe head injuries (chronic). Increased vessel fragility can lead to bleeding after minimal trauma. Interestingly, a 14‐year‐old patient with D‐2‐hydroxyglutaric aciduria showed loss of smooth muscle cells and fibrosis with deposition of fibrillary collagen in the cerebral microvessels, suggesting universal vessel‐wall pathology as part of this condition.^22^ Remarkably, our patient had a rapid progression of macrocephaly (head circumference increased >2 cm in a month), which was postulated to be secondary to increased intracranial pressure (ICP) from the space occupying subdural hygromas. The infant skull can expand to accommodate the increased volume, so that ICP is only minimally elevated or normal, without any clinical symptoms that can be seen with more severe ICP increases. Few reports in the literature describe similar findings in D‐2‐hydroxyglutaric aciduria patients: a 7‐month‐old boy with macrocephaly and bilateral subdural hemorrhages, cerebral atrophy and periventricular hypodensities,^23^ and a 4‐month‐old girl presented with bilateral hygromas and multiple retinal hemorrhages.^24^ Both were suspected of NAT, before the neurometabolic condition was diagnosed. Thus, we suggest that D‐2‐hydroxyglutaric aciduria should be included in the list of metabolic conditions (cobalamin C defect,^25^ bile salt transport defect,^3^ biliary atresia,^26^ primary or secondary vitamin K deficiency,^27^ glutaric aciduria type I,^6^ and Menkes disease^5^) that should be considered in the differential diagnosis of NAT. It should also be remembered that the diagnosis of a metabolic condition does not exclude nonaccidental injury. The clinical distinction between a medical diagnosis and NAT may be extremely challenging as infants with serious medical conditions are often irritable and demand greater parental care, ultimately putting them at higher risk of NAT.^12^ Thus, there is a need for a complete, careful, and nonjudgmental evaluation for NAT so that the correct diagnosis (medical condition, NAT, or both) can be reached in a timely fashion. We propose that infants with suspected NAT should be investigated for metabolic disorders, using urine organic acid analysis, in addition to the standard evaluation of nonaccidental trauma. Our case highlights the importance of a coordinated response by a broad spectrum of clinical and laboratory expertise, including Clinical Genomics, Child Neurology, Primary Care, Child Abuse Pediatrics, Neurosurgery, Radiology, and Biochemical Genetics. Only then metabolic disorders with unusual manifestations that can mimic nonaccidental trauma will be recognized, avoiding devastating consequences for patients and their families.

## 4 CONCLUSION

Several metabolic diseases, including D‐2‐hydroxyglutaric aciduria type I, can present with symptoms characteristic of nonaccidental trauma, like subdural and retinal hemorrhages. It is critical that care teams evaluate cases of suspected NAT with a methodical and nonjudgmental approach to ensure these rare genetic conditions are not overlooked.

## CONFLICT OF INTEREST

Ester Perales‐Clemente, Angela L. Hewitt, April L. Studinski, Jan‐Mendelt Tillema, William J. Laxen, Devin Oglesbee, Arne H. Graff, Piero Rinaldo, and Brendan C. Lanpher declare that they have no conflict of interest.

## AUTHOR CONTRIBUTIONS

Ester Perales‐Clemente, Angela L. Hewitt, Jan‐Mendelt Tillema, Piero Rinaldo, Arne H. Graff, and Brendan C. Lanpher contributed pertinent aspects of the planning, conduct, and reporting of the work described in the article. All authors were involved in drafting the article or revising it critically for important information, and all agreed to submission.

## INFORMED CONSENT

This article does not contain any studies with human or animal subjects performed by the any of the authors.
